# Transcriptome Analyses Provide Insights into the Aggressive Behavior toward Conspecific and Heterospecific in *Thitarodes xiaojinensis* (Lepidoptera: Hepialidae)

**DOI:** 10.3390/insects12070577

**Published:** 2021-06-25

**Authors:** Zhongchen Rao, Li Cao, Hua Wu, Richou Han

**Affiliations:** 1 Guangdong Key Laboratory of Animal Conservation and Resource Utilization, Guangdong Public Laboratory of Wild Animal Conservation and Utilization, Institute of Zoology, Guangdong Academy of Sciences, Guangzhou 510260, China; raozc@giz.gd.cn (Z.R.); caol@giabr.gd.cn (L.C.); wuhuaflower@126.com (H.W.); 2 Department of Plants and Crops, Faculty of Bioscience Engineering, Ghent University, 9000 Ghent, Belgium

**Keywords:** transcriptome analysis, aggressive behavior, *Thitarodes xiaojinensis*, gene expression, WGCNA

## Abstract

**Simple Summary:**

Aggression is an evolutionarily conserved, complex behavior, essential for survival, reproduction, and the organization of social hierarchies. It is well studied in adult insects, such as flies, ants, honey bees, and crickets. However, the study of aggressive behavior in the larval stage is still lacking. *T. xiaojinensis* is a common species found in mountainous regions of the Tibetan Plateau, the larvae of which are highly aggressive toward conspecifics. High-throughput RNA-seq with a reference genome provides opportunities for in-depth analysis when *T. xiaojinensis* is aggressive toward conspecifics and heterospecifics. This study provided a set of important pathways and DEGs associated with aggressive behavior. We also constructed the weighted gene co-expression network for traits, and the central and hub genes involved in aggressive behavior were obtained. The results revealed the molecular responses when *T. xiaojinensis* showed aggressiveness toward conspecifics and heterospecifics. These data are important for better understanding the aggressive behavior of Lepidopteran larvae at the transcriptional level and provide a theoretical basis for the further analysis of the genetic mechanism of the insect’s aggression.

**Abstract:**

Aggressive behavior in animals is important for survival and reproduction. It is well studied in adult insects, such as flies, ants, honey bees, and crickets. However, the larvae of Lepidopteran insects are also aggressive, studies of which are still lacking. Here, RNA-seq was used to generate a high-quality database for the aggressive behavior of *Thitarodes xiaojinensis* toward conspecifics and heterospecifics. Although there was similar aggressive behavior between the conspecific group and heterospecific group, significant differences were identified at the transcriptional level. When there was aggressive behavior toward conspecifics, *T. xiaojinensis* trended toward higher expression at the respiratory chain, while cuticle development and metabolism may have interfered. On the other hand, when there was aggressive behavior toward *H. armigera*, genes related to neuron and cuticle development, cellular processes, and its regulated signaling pathways were significantly upregulated, while the genes associated with oxidation-reduction and metabolism were downregulated. Weighted gene co-expression networks analysis (WGCNA) was performed, and two modules with properties correlating to the aggressive behavior of *T. xiaojinensis* were identified. Several hub genes were predicted and confirmed by qRT-PCR, such as *CLTC*, *MYH*, *IGF2BP1*, and *EMC*. This study provides a global view and potential key genes for the aggressive behavior of *T. xiaojinensis* toward conspecifics and heterospecifics. Further investigation of the hub genes would help us to better understand the aggressive behavior of insects.

## 1. Introduction

Aggressive behavior occurs in many species, such as fish, frogs, lizards, insects, and most mammals, including humans 1. Aggression is a near-universal animal behavior, and it plays important roles in obtaining and defending food, progeny, and mates; avoid-ing and defending against predators; and establishing and maintaining stable social hier-archies in some animals.

Aggressive behavior is a complicated, quantitative trait, with population variation due to multiple interacting loci, with small individual effects, whose expression depends on the physical and social environment. Aggressive behavior is an evolutionarily conserved complex behavior from mammals to insects; a range of studies have explored some of the underlying mechanisms regulating aggression by examining the role of sensory systems [2,3], sex-determining pathways [4,5], the nervous system [6,7], and biogenic amines [8,9,10]. Both of the olfactory sensory neurons expressing receptors *Or65a* [11] and *Or67d* [12] could detect the male-specific volatile pheromone 11-cis-vaccenyl acetate (cVA), involved in promoting aggression in solitary males and suppressing aggression in group-housed males, respectively, associated with promoting aggression in isolated males and suppressing aggression in group-housed males [13,14]. Gustatory receptor neurons expressing *Gr32a* could modulate the male–male aggression triggered by cuticular hydrocarbon(z)-7-tricosene [2], while another gustatory receptor *Gr63a*, encoding a subunit of the receptor for CO_2_, were also implicated in aggressive behavior [15]. In male files, conditional activation of P1 neuron subsets induced immediate courtship behavior and long-term aggressive behavior. This process was double-negative regulation concerning both fruitless (*Fru*) and doublesex (*Dsx*) neurons in the P1 cluster [10,16], where the *Fru* neurons regulate courtship and the *Dsx* neurons control aggression [4]. To date, many studies have used the candidate gene approach to identify the role of neuro-transmitters in meditating and regulating aggression levels. In particular, biogenic amines and the genes affecting their biosynthesis and metabolism are implicated in aggressive behavior in mammals [17,18] and invertebrates [3,3,19,20,21]. γ-aminobutyric acid, the neuro-transmitter nitric oxide, and the neuropeptide Y affect aggressive behavior in mammals [22,23,24]. Neuropeptide Y affects aggression in mammals [25,26]. Neuropeptide F, the invertebrate homolog of neuropeptide F, affects aggression in *Drosophila* [27]. In addition to the well-characterized pathways mentioned above, more novel genes affecting aggressive behavior have been identified, including the cytochrome P450 gene family [28] and genes associated with electron transport, voltage-gated potassium channel, catabolism, G-protein coupled receptor signaling, and basic cellular and metabolic processes [9,29,30].

In previous studies, aggressive behavior was widely investigated in adult insects, such as *D. melanogaster* (Diptera: Drosophilidae), *Nylanderia fulva* (Hymenoptera: Formicidae), *Apis mellifera* (Hymenoptera: Apidae), and *Gryllus bimaculatus* (Orthoptera: Gryllidae) [9,15,31,32,33], while there were seldom studies on the larvae of Lepidopteran. However, aggressive behavior frequently occurs in Lepidopteran larvae. *Thitarodes xiaojinensis* (Lepidoptera: Hepialidae) is a flag species of the Tibetan Plateau, and inhabit the soil layer 50–200 mm below the surface (4100–4600 m). The larva of *T. xiaojinensis* is one of the host species of the caterpillar fungus complex, *Ophiocordyceps sinensis*, which is used in traditional Chinese medicine with important economic and medical values. Artificial cultivation of the caterpillar fungus complex is urgently needed to ensure its protection as a bio-resource and for commercial supply [34]. One of the crucial steps in the artificial cultivation of this complex is the artificial rearing of the insect hosts. However, the larvae of insect hosts, such as *T. xiaojinensis,* cannot be reared in groups in the laboratory, because they exhibit aggressiveness toward conspecifics. The *Thitarodes* species is a long lifespan holometabolous insect with four developmental stages, including approximately 38–53 days in the egg stage, 218–796 days in the larval stage, 35–41 days in the pupal stage, and 3–8 days in the adult stage in laboratory conditions [34]. In the wild, their larval stage would be much longer, as they need to hibernate in the winter [35]. Another example of an aggressive Lepidopteran insect is *Helicoverpa armigera* (Lepidoptera: Noctuidae), the larva of which show aggressive and even cannibalistic behavior toward conspecifics [36]. Both insect larvae display aggressive behavior toward each other during encountered.

In this study, we focus on the aggressiveness of *T. xiaojinensis* toward conspecific *H. armigera* at the transcriptional level. The *T. xiaojinensis* larvae in the solitary group (one *T. xiaojinensis* larva), conspecific group (two *T. xiaojinensis* larvae), and heterospecific group (*T. xiaojinensis* larva and *H. armigera* larva) were examined by RNA-seq technology. These results provide interesting information for understanding the molecular responses of aggressive behavior in insect larvae when encountering conspecifics and heterospecifics.

## 2. Materials and Methods

### 2.1. Experimental Insects and Assays

*T. xiaojinensis* pupae were collected from alpine meadows in Xiaojin, Sichuan, China, and reared on carrots (*Daucus carota*) in Guangzhou, Guangdong, China, under laboratory conditions of 13–16 °C, 60–80% relative humidity (RH), and 16 h:8 h light:dark photoperiod. Every year, new individuals collected at the same locations were added to the laboratory population to avoid inbreeding and loss of genetic variability. The insect species was identified as *T. xiaojinensis* by using the amplified cytochrome b sequence with the primers CB1 (TATGTACTACCATGAGGACAAATATC) and CB2 (ATTACACCTCCTAATTTATTAGGAAT) [34,37]. The *H. armigera* larvae and artificial diet were purchased from a commercial producer (Jiyuan Baiyun Industry Co. Ltd., Jiyuan, Henan province, China) and reared in the laboratory at 25–30 °C, 60–70% RH.

### 2.2. Laboratory Trials

#### 2.2.1. Experimental Design and Procedures

Healthy 2nd, 4th, and 6th instar larvae (L2, L4, and L6) of *T. xiaojinensis* and 5th instar larvae (L5) of *H. armigera* were selected for the experiment. The *T. xiaojinensis* larvae with similar weight (L2: 0.05 ± 0.01 g; L4: 0.4 ± 0.03 g; L6: 0.66 ± 0.03 g) in the same instar were selected. The L5 *H. armigera* (3.5 ± 0.3 cm) with a similar length to L6 *T. xiaojinensis* (3.8 ± 0.3 cm) were selected. All individuals were isolated in a 3.5 cm (diameter) container and starved for seven days (for *T. xiaojinensis*) and one day (for *H. armigera*) before use in the assays. Individuals were then transferred to a new container in pairs, depending on the assays described below.

##### Assay for Testing Aggressive Behavior

For testing the aggressive behavior of *T. xiaojinensis* toward conspecifics, nine comparing groups were determined; L2–L2 (two L2 larvae caged together, one of the larvae was marked and scored), L2–L4 (L2 larva caged with L4 larva, L2 larva was scored), L2–L6, L4–L2 (L2 larva caged with L4 larva, L4 larva was scored), L4–L4, L4–L6, L6–L2, L6–L4, and L6–L6. A total of six combinations were observed (Figure S1), including L2 vs. L2 combination; L2 vs. L4 (including L2–L4 and L4–L2 comparing groups), L2 vs. L6 (including L2–L6 and L6–L2 groups), L4 vs. L4, L4 vs. L6 (including L4–L6 and L6–L4 groups), and L6 vs. L6 combinations; all combinations were performed in a 3.5 cm container. The combinations were observed for the first 10 min of an introduced social interaction. The following behaviors, after they encountered, were defined as aggressive behavior:

- Hit (by head or tail);
- Bite (by jaw);
- Chase (the attacker pursues the victim).

When the above behaviors were observed, one point was added to the corresponding comparing group, and the total point of the comparing group was defined as its aggressive behavior scale (ABS). For example, if L4 larva hit/bite/chase the L6 larva in the L4 vs. L6 combination, one point was added to the L4–L6 comparing group, on the other hand, if L6 larva hit/bite/chase the L4 larva in the L4 vs. L6 combination, one point was added to L6–L4 comparing group.

For testing the aggressive behavior of L6 *T. xiaojinensis* toward L5 *H. armigera*, four comparing groups were determined: L6–L6, L6–L5 (L6 *T. xiaojinensis* larva caged with L5 *H. armigera* larva, L6 *T. xiaojinensis* larva was scored), L5–L5, and L5–L6. In total, three combinations were observed, L6 vs. L6, L5 vs. L5, L5 vs. L6 (including L6–L5 and L5–L6 comparing groups) (Figure S1). Each combination was performed in a 3.5 cm container. The observation time and scored rule were the same as mentioned above.

##### Assay for Testing Survival Rate

The comparing groups and combinations were the same as the assay for testing aggressive behavior. Survival time was recorded in this assay. When one of the larvae in the group died, the time point was marked as “death event”. The trials were performed over 72 h; each container was observed every 3 h until the twelfth hour of the first day, and then the trial was performed again on the second day and the third day.

Both assays included two conditions—feeding or starving. Thirty repetitions were performed for each combination, and, in total, eight combinations were tested (assay 1: L2 vs. L2, L2 vs. L4, L2 vs. L6, L4 vs. L4, L4 vs. L6, L6 vs. L6 (shared with assay 2); assay 2: L6 vs. L6, L5 vs. L5 and L6 vs. L5) (Figure S1). The assays were conducted under 16 ± 1 °C, 60–70% RH, and a light photoperiod.

#### 2.2.2. Statistical Analysis

For the data that were not normally distributed (by Shapiro–Wilk normality test, *p* = 8.81 × 10^−4^), and the variances of which were heterogeneity (by Bartlett test of homogeneity of variances, *p* = 5.49 × 10^−5^), we used the Kruskal–Wallis H test to determine the statistical differences for multiple comparisons among more than two groups, followed by the Nemenyi test for two-group comparisons. The analysis was performed using the “pgirmess” package from R software (4.0.2). The survival analysis (accumulate survival probability) of different comparing groups was performed with the Kaplan–Meier procedure [38,39]. This procedure is a method to estimate the time-to-event method in the presence of censored cases. Within the Kaplan–Meier procedure, the equality of survival function was compared with the log-rank test [40] using the “survival” and “survminer” packages from R software (4.0.2), and the relating plots were generated by the “ggfortify” and “ggplot2” packages from R software (4.0.2).

### 2.3. RNA-Seq Experiment

#### 2.3.1. Library Construction

Healthy L2, L4, and L6 *T. xiaojinensis* were selected for RNA-seq. Nine groups were determined, including 1) three solitary groups: unique L2 (TS2), L4 (TS4), and L6 (TS6) *T. xiaojinensis* larva used as control; 2) three conspecific groups: L2–L2 (TG2), L4–L4 (TG4), and L6–L6 (TG6) *T. xiaojinensis* larvae; 3) one heterospecific group (L6 *T. xiaojinensis* caged with L5 *H. armigera*): L6 *T. xiaojinensis* larva (TM6).

After 7 days of starvation, all groups were tested in a 3.5 cm container. The size of the container ensured the adequate contact of two larvae (easily encounter and have enough place to retreat). In the TG2, TG4, TG6, and TM6 groups, the experiment did not end until one of the larvae showed aggressive behavior (hit or bite) toward the partner. The attacker was selected and the whole body was immediately frozen in liquid nitrogen, and stored at –80 °C, to maintain the transcriptional status in a specific state. The victim was abandoned.

#### 2.3.2. Preparation of RNA, Library Construction, and Sequencing

Total RNA was extracted with TRIzol, according to the manufacturer’s instructions. A NanoDrop ND-2000 spectrophotometer, non-denaturing agarose gel electrophoresis, Qubit 2.0, and Agilent 2100 Bioanalyzer (Agilent, Santa Clara, CA, USA) were used to determine the quantity and quality of RNA in the samples. A total of 21 individual cDNA libraries were constructed from solitary samples (TS2, TS4, and TS6); conspecific samples (TG2, TG4, and TG6); and heterospecific samples (TM6). Each sample with 3 biological replicates and 10 individuals was used for each biological replicate. The quantification and qualification of the libraries were analyzed on Qubit 2.0, Agilent 2100 Bioanalyzer (Agilent, Santa Clara, CA, USA), and ABI StepOnePlus Real-Time PCR system (ABI, Waltham, MA, USA). An Illumina NovaSeq 6000 platform (Illumina, San Diego, CA, USA) was used for sequencing. All of the raw sequence data were deposited in the NCBI Sequence Read Archive (SRA) under BioProject accession number PRJNA657440. 

#### 2.3.3. Mapping and Transcriptome Annotation

The reads that contained adapter sequences with more than 10% uncertain base pairs, and with low quality, were removed. The resulting clean reads were used to perform quality control through base composition and quality distribution. Only the clean reads with a balanced composition, as well as high distribution of high-quality base (sequencing quality value >20), were kept. The remaining clean reads were mapped to the genome of *T. xiaojinensis* using HISAT2 (2.0.6) [41]. StringTie (v1.0.4) was used to reconstruct transcripts [42], and the potential novel transcripts were predicted by cufflinks (v2.2.1) [43]. All of the novel transcripts were annotated against the NCBI non-redundant protein database and Swiss-Prot database using BLASTX (e-value < 1 × 10^−5^). 

#### 2.3.4. Differentially Expressed Gene Analysis

RSEM (v 1.2.31, RNA-seq by expectation maximization) [44], a utility package in the software Trinity, was used to estimate the abundance of transcripts and the fragments per kilobase per million mapped read (FPKM), which were calculated for the digital gene expression profile. DEGs were calculated using edgeR [45]. The *p*-values were corrected for multiple hypothesis tests, and the threshold *p*-value by False Discovery Rate (FDR) was determined. Genes in different samples with FDR < 0.05 and |fold change| > 2 were considered DEGs.

#### 2.3.5. Functional Enrichment Analysis

The Gene Ontology (GO) enrichment analysis was performed with the Database for Annotation, Visualization, and Integrated Discovery (DAVID v6.8) [46]. GO visualization was performed with the topGO (v2.40.0) package from R software [47]. Z-score is an easily calculated value that could provide an indication as to whether the BP/MF/CC is more likely to decrease (negative value) or increase (positive value). The calculated formula is as follows:

$$\frac{\left(Up-Down\right)}{\sqrt{Background}}$$

*Up* and *Down* are the number of assigned genes upregulated (log_2_FC > 1) or downregulated (log_2_FC < −1); *Background* is the number of genes assigned to a GO term.

Pathway enrichment analysis was performed using the KEGG Orthology Based Annotation System (KOBAS v3.0) [48], with a threshold *p*-value ≤ 0.05. DEGs were also analyzed by Gene Set Enrichment Analysis (GSEA v4.1.0) [49,50]. The GO and KEGG annotation of all genes in *T. xiaojinensis* were used as a gene sets database. The gene sets with FDR *q*-value < 0.05 were considered statistically significant.

### 2.4. WGCNA Analysis 

#### 2.4.1. Construction of Weighted Gene Co-Expression Network

The weighted gene co-expression network was constructed for the *T. xiaojinensis* data sets to identify gene modules associated with different expression patterns when *T. xiaojinensis* showed aggressive behavior toward conspecifics or heterospecifics, following the previously described algorithm [51]. The TG6-3 sample was abandoned, as it was considered an outlier by the WGCNA method. All expressed genes (9928 genes) from 20 *T. xiaojinensis* libraries with FPKM > 1 in at least one sample were selected for constructing the weighted co-expression network, by the R package WGCNA [52]. The scale independence and average connectivity of different power modules (power: 1–20) were tested by the gradient independence method. Based on the scale-independent conditions, the signed R^2 was set to 0.85 and the power value (β) was determined. The gene modules were constructed, and the genetic information corresponding to each module was extracted. To ensure the reliability of the results, the minimum number of genes per module was set to 30.

#### 2.4.2. Interactions Analysis of Co-Expression Modules

We calculated the interaction relationship between different co-expression modules, and constructed the topological overlap matrix (TOM) by using the correlation expression values [51]. The hierarchical cluster analysis was performed with the FlashClust function, using each TOM as input. Then, the module was detected by the DynamicTreeCut algo-rithm, and a branch of the dendrogram was generated [52]. The color of the modules was assigned randomly, and the module characteristic gene (eigengene) of each module was calculated by the first principal component. The module eigengene represents the gene expression pattern in this module, which merges highly relevant eigengenes (mergeCutHeight = 0.2). The Pearson correlation between the module eigengenes and the interest characteristics was calculated, the module–trait relationship was estimated by us-ing the module eigengenes, and the classification of samples was based on the corre-sponding traits. The modules with a correlation coefficient ≥ |0.75| and *p*-value ≤ 1e-5 were selected for subsequent analysis. A heatmap of the gene network topological overlap was used to visualize the structure of the co-expression module. In addition, the relation-ship between the modules was summarized by the hierarchical clustering of the module eigengenes and the eigengene adjacency heatmap [52].

#### 2.4.3. Identification of Hub Genes

Module membership (MM) represents the correlation of the expression of each gene to corresponding module eigengenes. The higher the MM, the greater the role that the gene plays in the corresponding module. Gene significance (GS) represents the correlation be-tween each gene and the trait of interest [52]. The genes with the highest MM and GS were selected as candidate genes for further analysis in the interested module [53]. In this study, genes with an MM > 0.8 and GS > 0.6, with a threshold of *p*-value < 0.05, were defined as central genes. The central genes were used for subsequent analysis. The sum of the correla-tion coefficients with other nodes in the corresponding module of one gene was the intra-modular connectivity of this gene [54]. The genes with higher intramodular connectivity also played more important roles in the corresponding module. The hub gene represented a highly connected gene, with a high degree of connectivity in co-expression modules. Based on the size of the module, the top 50 (for the Turquoise module) or 10 (for other modules) of the genes with the highest intramodular connectivity within each module were referred to as intramodular hub genes. We constructed and visualized the co-expressed networks using Cytoscape 3.0 [55].

### 2.5. Quantitative Real-Time PCR

RNA from the larvae of *T. xiaojinensis* in different groups was used to validate the RNA-seq experiment. RNA extracted from ten individuals from the same groups was mixed as one replicate. A total of 1 μg of RNA from the transcriptome sample was used for cDNA synthesis, according to the manufacturer’s protocol (PrimeScript™ RT Reagent Kit with gDNA Eraser, TaKaRa, Japan). The 25 μL reaction consisted of 2 μL of diluted cDNA (1:2), 12.5 μL of SYBR® Premix Ex Taq II (Tli RNaseH Plus) (TaKaRa, Japan), and 0.2 mM of each primer, and these were used for the qRT-PCR reaction. All reactions were performed on a Stratagene MX3000P qPCR system (Stratagene, Santa Clara, CA, USA), according to the manufacturer’s instructions. Thermal cycling conditions were set to 95 °C for 1 min of initial denaturation, followed by 40 cycles of 95 °C for 15 s, 60 °C for 30 s, and 72 °C for 30 s of amplification. Then, a melting curve analysis from 55 °C to 95 °C was applied to all reactions to ensure consistency and specificity of the amplified product. All primers used for the testing genes are described in Table S1. Quantitative measurements were normalized by the reference gene ribosomal protein S3 and glyceraldehyde-3-phosphate dehydrogenase, and relative expression levels were calculated using the 2^-ΔΔCt^ method [56,57]. In the regression analysis, and the fold changes of RNA-seq were base-2 logarithm of FPKM ratios (TG6-3 sample was abandoned), and the fold changes of qRT-PCR were ΔΔCt [56].

## 3. Results

### 3.1. Behavior Observation

To understand the aggressiveness of *T. xiaojinensis* toward conspecifics, L2, L4, and L6 larvae were chosen for observation. Feeding and starved conditions were separated. Thirty repetitions were performed for each combination. For *T. xiaojinensis*, hunger tolerant insects, a suitable starvation treatment time had to be selected. After 7 days of starvation, the consumption rate of carrot was significantly higher than that of 1-day and 3-day starvation (Figure S2), and a “consumption event” was observed at all samples at 7 days for testing. Therefore, 7 days without feeding was considered a state of starvation for *T. xiaojinensis*.

#### 3.1.1. Aggressive Behavior of *T. xiaojinensis* toward Conspecific

**Figure 1:**
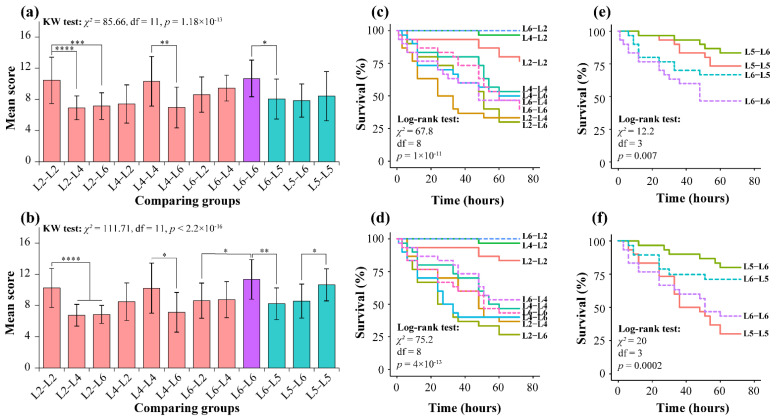
Statistical analysis of aggressive behavior and Kaplan–Meier analysis for different assays. Mean aggression behavior scale (ABS) of different comparing groups with food (**a**) or without food (**b**). Salmon, turquoise, and violet bars represent conspecific groups, heterospecific groups, and the group share in conspecifics and heterospecifics, respectively. The Kruskal–Wallis H test was used for overall group comparisons, followed by the Nemenyi test for two-group pairwise comparisons: ns, *p* > 0.05; *, *p* < 0.05; **, *p* < 0.01; ***, *p* < 0.001; ****, *p* < 0.0001. The survival rate for conspecifics of *T. xiaojinensis* when providing food (**c**) or without food (**d**). The survival rate for *H. armigera* and *T. xiaojinensis* caged with conspecifics or heterospecifics when providing food (**e**) or without food (**f**). The “death event” was marked when one of the events was death. The log-rank test was performed to yield significance for overall groups. L2, L4, and L6 represent 2nd, 4th, and 6th instar *T. xiaojinensis* larvae, respectively; L5 denotes 5th instar *H. armigera*. The observed object in the group is shown before the hyphen. For example, for the L2–L4 group, the combination of L2 vs. L4 focuses on L2; for the L4–L2 group, the combination of L2 vs. L4 focuses on L4.

In the feeding condition, significantly different ABS scores were found among nine groups (KW test, *χ^2^* = 85.49, df = 8, *p* = 3.81 × 10^−15^) (Table S2). For L2 larvae, ABS scores were significantly higher in the L2–L2 group than in the L2–L4 (Nemenyi test, *p* < 0.0001) and L2–L6 (*p* < 0.001) groups. For L4 larvae, ABS scores were significantly higher in the L4–L4 group than in the L4–L6 group (*p* < 0.01). Similar ABS scores were found among the L6–L2, L6–L4, and L6–L6 groups (Figure 1a). Moreover, no differences in ABS scores were found among L2–L2, L4–L4, and L6–L6, implying similar aggressiveness in *T. xiaojinensis* toward the same instar larvae under the feeding condition. The aggressive behavior under the starved condition was similar to that under the feeding condition. Significantly different ABS scores were also found among nine groups (KW test, *χ^2^* = 90.8, df = 8, *p* < 3.18 × 10^−16^) (Table S2). Moreover, the significantly different groups were L2–L2 to L2–L4 (*p* < 0.0001) and L2–L6 (*p* < 0.0001), L4–L4 to L4–L6 (*p* < 0.05), and L6–L6 to L6–L2 (*p* < 0.05) groups (Figure 1b). Similarly, no differences in ABS scores were found among L2–L2, L4–L4, and L6–L6. A pairwise comparison was also performed between the feeding group and the starved group; no different aggressive behaviors were found under these two conditions (Table S2). Among the three aggressive behaviors, the “hit” behavior was much more frequent than the “bite” behavior, and the “chase” behavior rarely occurred, whether in the feeding or the starved condition (Figure S3 and Table S3).

In the feeding condition, the survival rate of L2 larvae was higher in the L2–L2 group (76.7 ± 7.7%) and then decreased in L2–L4 (33.3 ± 8.6%) and L2–L6 (30 ± 8.3%) (Figure 1c). Pairwise comparison showed that significant differences were found between L2–L2 and L2–L4 (*p* = 5.3 × 10^−4^), as well as L2–L2 and L2–L6 (*p* = 4.5 × 10^−4^), but similarities were noted between L2–L4 and L2–L6 (*p* = 0.62) (Table S4). Similar results were observed in the L4 and L6 groups in the feeding condition; the survival rate of the L4–L2 and L6–L2 groups was significantly higher than the other groups caged with older larvae (Figure 1c and Table S4). The survival rate under the starved condition is consistent with that in the feeding condition (Figure 1d). For the L2 larvae groups, the survival rate of L2 larvae was also significantly higher in the L2–L2 group compared with the L2–L4 and L2–L6 (*p* = 5.3 × 10^−4^, *p* = 2.3 × 10^−5^) groups. A similar trend was also found in L4 and L6 larvae groups under starved conditions (Table S4), indicating that the survival rate of *T. xiaojinensis* larva decreased along with an increasing larval instar of the partner. Pairwise comparison showed that no group was significantly different between the feeding and starved condition (*p*-value ranging from 0.43 to 1) (Table S4); thus, demonstrating that, together, with the results of aggressive behavior mentioned above, food has a limited influence on the aggressive behavior of *T. xiaojinensis* larvae. Notably, in addition to the aggressiveness, the survival rate was also affected by organ development, such as jaw (attack) and cuticle (defend); the older larvae may have a bigger jaw and thicker cuticle.

#### 3.1.2. *T. xiaojinensis* and *H. armigera* Larvae Caged with Same Instar Conspecifics or Similar-Sized Heterospecifics

To understand the aggressive behavior of *T. xiaojinensis* toward conspecifics or heterospecifics, we chose L6 *T. xiaojinensis* and L5 *H. armigera* larvae for observation. The assays also separated feeding and starved conditions. In the feeding condition, significant differences in ABS scores were found among four groups in the feeding (KW test, *χ^2^* = 19.04, df = 3, *p* = 2.68 × 10^−4^) or starved (KW test, *χ^2^* = 28.3, df = 3, *p* = 3.14 × 10^−6^) condition (Table S2). For *T. xiaojinensis*, the ABS scores of L6–L6 were significantly higher than that in L6–L5 (Nemenyi test, *p* < 0. 05) (Figure 1a). Similar results were found under the starved condition (*p* < 0.01) (Figure 1b), implying that *T. xiaojinensis* larvae showed higher aggressiveness toward the same instar conspecific than toward similar-sized heterospecifics, whether providing food or not. In addition, similar to the conspecific cage, the “hit” behavior was more frequent than the “bite” behavior when L6 *T. xiaojinensis* was caged with heterospecifics, and the “chase” behavior rarely occurred (Figure S3 and Table S3). On the other hand, different from *T. xiaojinensis*, although similar ABS scores were found between L5–L5 and L5–L6 groups under feeding conditions, significant differences were found under starved conditions (*p* < 0.05) (Table S2), suggesting that food was associated with aggressive behavior in *H. armigera*. Moreover, it appeared that no significantly different aggressive behaviors were found between L6–L6 and L5–L5 groups, whether in feeding or starved conditions (Figure 1a,b, Table S2).

In the feeding condition, when caged with conspecifics, the survival rates of *T. xiaojinensis* (L6–L6, 46.7 ± 9.1%) and *H. armigera* (L5–L5, 73.3 ± 8%) were quite different (*p* = 0.046) (Figure 1e and Table S4), and the lower survival rate of *T. xiaojinensis* toward conspecifics may demonstrate the higher aggressiveness of *T. xiaojinensis* under feeding condition. When caged with heterospecifics, the survival rates of *T. xiaojinensis* and *H. armigera* were 66.7 ± 8.6% (L6–L5) and 83.3 ± 6.8% (L5–L6), respectively (Figure 1e). Pairwise comparison showed no significant differences between them (*p* = 0.18) (Table S4). Moreover, the survival rates of those caged with conspecifics compared to heterospecifics were calculated. For *T. xiaojinensis*, the survival rate was higher when caged with heterospecifics (L6–L5 vs. L6–L6: 66.7 ± 8.6% vs. 46.7 ± 9.1%, *p* = 0.24), similarly to the results for *H. armigera* (L5–L6 vs. L5–L5: 83.3 ± 6.8% vs. 73.3 ± 8%, *p* = 0.51). Both insects showed no significant differences when caged with conspecifics and heterospecifics (Table S4). In the starved condition, when caged with conspecifics, the survival rates were similar (*p* = 0.58), but the values of *T. xiaojinensis* (L6–L6, 43.3 ± 9 %) were higher than that of *H. armigera* (L5–L5, 30 ± 8.3%), which was different from that in the feeding condition (Figure 1f and Table S4). When caged with heterospecifics, similar survival rates were found between *H. armigera* and *T. xiaojinensis* (*p* = 0.57); the values were 73.3 ± 8% (L6–L5) and 80 ± 7.3% (L5–L6), respectively (Table S4). Additionally, the survival rates of those caged with conspecifics compared to heterospecifics were also calculated. For *T. xiaojinensis*, the survival rate of L6 larva caged with conspecifics (L6–L6) was higher than for those caged with heterospecifics (L6–L5) (L6–L5 vs. L6–L6: 73.3 ± 8% vs. 43.3 ± 9%, *p* = 0.05). Similarly, in *H. armigera*, the group caged with heterospecifics (L5–L6: 80 ± 7.3%) had a significantly higher survival rate than those caged with conspecifics (L5–L5: 30 ± 8.4%, *p* = 0.001) (Table S4). The results reveal that both insects showed higher aggressiveness toward conspecifics rather than heterospecifics under starved conditions.

On the other hand, different survival rates were observed in *H. armigera* caged with conspecifics under different conditions (*p* = 0.004); a significantly lower value was found under starved conditions compared with feeding conditions (feeding: 30 ± 8.4%, starved: 73.3 ± 8%, *p* = 0.005). In contrast to *H. armigera*, *T. xiaojinensis* showed similar values when caged with conspecifics under both conditions (feeding: 46.7 ± 9.1%, starved: 43.3 ± 9%, *p* = 0.92) (Table S4). Together with the results of the ABS scores (Figure 1a,b), this suggests that food was a key factor in determining the aggression of *H. armigera* toward conspecifics, with little influence on *T. xiaojinensis*. Moreover, both insects had similar ABS scores (L6–L5: *p* > 0.05, L5–L6: *p* > 0.05) and survival rates (L6–L5: *p* = 0.63, L5–L6: *p* = 0.78) when caged with heterospecifics under both conditions (Tables S2 and S4). Exhibiting the target prey was another prior factor in determining the aggression of *T. xiaojinensis* and *H. armigera*.

### 3.2. RNA-Sequencing Analysis

**Figure 2:**
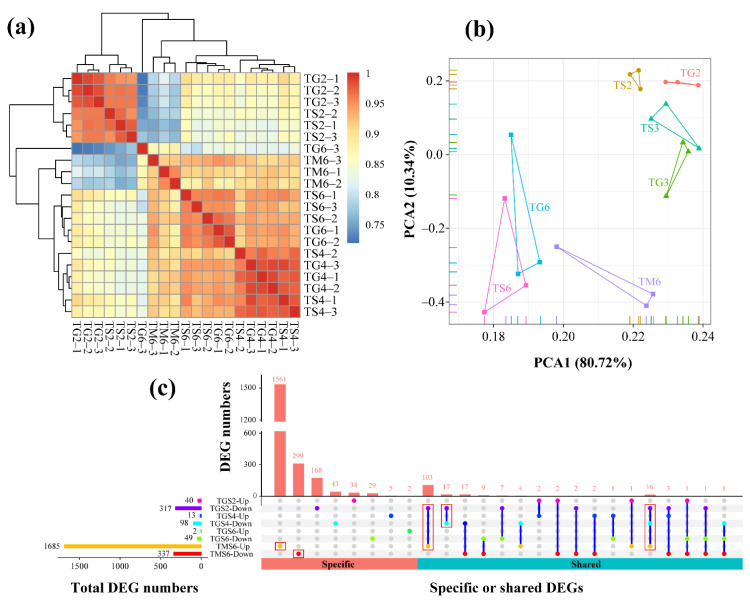
Correlation and differential gene expression analysis. (**a**) Heatmap of duplicate samples of different comparing groups of *T. xiaojinensis*. The color spectrum, ranging from blue through yellow to red, represents Pearson correlation coefficients ranging from 0.75 to 1, indicating low to high correlations. (**b**) Principal component analysis (PCA) of the transcriptome for different comparing groups of *T. xiaojinensis*. Different comparing groups are shown in different colors; *T. xiaojinensis* of different instars are indicated by different symbols. (**c**) UpSetR plots depict specific and shared DEGs among different comparing groups from *T. xiaojinensis*. Horizontal bars represent the total DEG numbers of each comparing group; vertical bars represent the unique and shared DEGs of different comparing group intersections. TGS2, TS2 vs. TG2; TGS4, TS4 vs. TG4; TGS6, TS6 vs. TG6; TMS6, TS6 vs. TM6.

Whole-genome mRNA sequencing was used to monitor changes in gene expression in *T. xiaojinensis* when there was aggressive behavior toward conspecifics and *H. armigera*. In total, 232.38 Gb high-quality clean data were obtained from *T. xiaojinensis* (Table S5). A total of 21 libraries were constructed, including nine solitary samples (TS2, TS3, and TS6), nine samples from conspecific groups (TG2, TG3, and TG6), and three samples from the heterospecific group (TM6), with at least 60.97% clean reads matching to the corresponding genome, using HISAT2 (Table S5). The correlation coefficient between the biologically replicated samples is shown in Figure 2a. The correlation coefficient between different samples in the same comparing group always exceeded 0.94. L2 and L3 samples from *T. xiaojinensis* were higher than the values between the different comparing group samples. Similar results were obtained in the principal component analysis (PCA) (Figure 2b). The FPKM value of expressed genes in different samples is shown in Table S6.

#### 3.2.1. Differential Gene Expression Analysis

To gain insights into global transcriptional changes in *T. xiaojinensis* larvae, in different stages and different treatments, pairwise comparisons were performed between the solitary and conspecifics/heterospecifics. A total of 2330 DEGs were identified in *T. xiaojinensis* from four comparing groups (|log_2_FC| > 1 and FDR < 0.05), including 357, 111, 51, and 2022 DEGs from comparing groups TS2 vs. TG2 (TGS2), TS4 vs. TG4 (TGS4), TS6 vs. TG6 (TGS6), and TS6 vs. TM6 (TMS6), respectively (Table S7). A higher number of downregulated genes was identified in conspecific groups: TGS2 (40 vs. 317), TGS4 (13 vs. 98), and TGS6 (2 vs. 49). Meanwhile, the heterospecific group showed the opposite. The number of upregulated genes of TMS6 was five-times higher than that of downregulated (1685 vs. 377) (Figure 2c), indicating that transcriptional responses vary greatly during the aggressive behavior of *T. xiaojinensis* toward *H. armigera*. The specific or shared DEGs among comparison groups were examined using the UpSetR diagram (Figure 2c). Moreover, 1561 out of 1685 genes (92.64%) were specifically upregulated in TMS6, while 299 (88.72%) were specifically downregulated (Figure 2c). A total of 33 genes were downregulated in both TGS2 and TGS4, but no up- or downregulated genes were shared among three conspecific groups (TGS2, TGS4, and TGS6). Notably, 103 genes were downregulated in TGS2, but upregulated in TMS6 (Figure 2c). A complete list of significant DEGs is provided in Table S7.

#### 3.2.2. Molecular Responses of *T. xiaojinensis* Larvae When Caged with Conspecific of the Same Instar Stage

**Figure 3:**
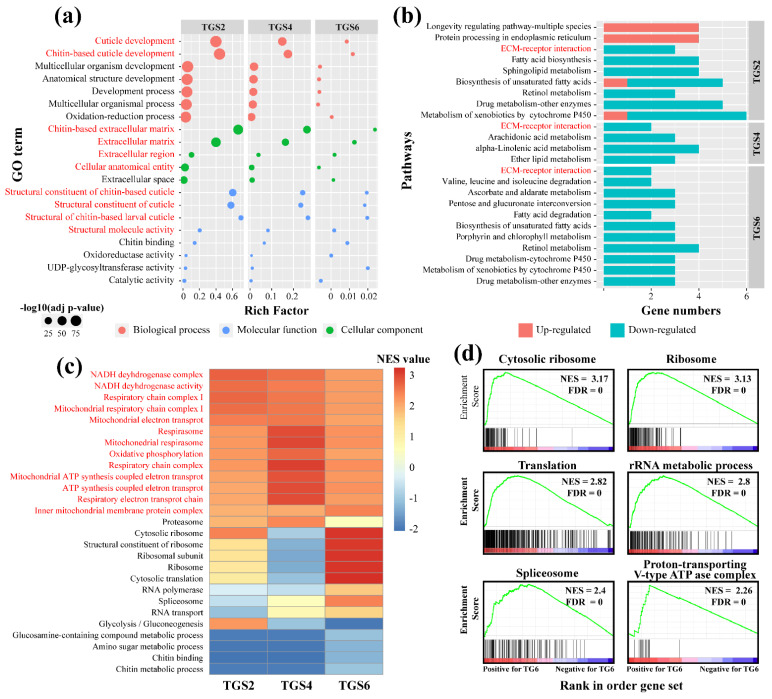
Functional enrichment analysis for TGS2, TGS4, and TGS6. (**a**) GO and (**b**) KEGG pathway enrichment for TGS2, TGS4, and TGS6 groups. All GO terms were enriched by downregulated genes. GO terms or pathways shared among TGS2, TGS4, and TGS6 groups are indicated by the red font. (**c**) Heatmap for GSEA analysis. The normalized enrichment score (NES) was used as input. Gene sets enriched in three comparing groups are indicated by the red font. (**d**) GSEA analyses of gene sets for TGS6. NES, normalized enrichment score. FDR, false discovery rate. The top portion of the plot shows the running ES for the gene set as the analysis moves down the ranked list. The bottom portion of the plot shows where the members of the gene set appear in the ranked list of genes. Positive (red) and negative (blue) NES indicate higher and lower expression in TGS samples, respectively.

The GO annotation statistics were performed on the DEGs in pairs; the results are shown in Table S8. For the TGS2 group, 102 GO terms were assigned. The biological processes (BP) of cuticle development (GO:0042335), chitin-based cuticle development (GO:0040003), cellular component (CC) of chitin-based extracellular matrix (GO:0062129), and molecular function (MF) of structural constituent of cuticle (GO:0042302) and structural molecular activity (GO:0005198) were well-presented, all of which were involved in cuticle development and were significantly suppressed in TGS2 groups. For the TGS4 and TGS6 groups, 69 and 20 GO terms were assigned, respectively; similar to the TGS2 group, the cuticle development-related processes were also significantly enriched and suppressed (Figure 3a). Moreover, all DEGs were mapped to a reference pathway in the KEGG database to determine the biological pathways in which these genes may have been involved (Table S9). For the TGS2 group, downregulated genes were enriched in various metabolic processes, including fatty acid biosynthesis, biosynthesis of unsaturated fatty acid, retinol metabolism, and drug metabolism. Similarly, the downregulated genes from the TGS4 and TGS6 groups were also enriched in metabolic processes, such as arachidonic acid metabolism, ether lipid metabolism, and fatty acid metabolism (Figure 3b). It seemed that when *T. xiaojinensis* showed aggressive behavior toward the same instar conspecific, cuticle development and metabolic processes were significantly suppressed, whether younger larvae or older larvae. GSEA results showed that oxidative phosphorylation and respiratory chain-related genes in *T. xiaojinensis* trended toward higher expression when there was aggressiveness toward the same instar stage conspecific (TG2, TG4, and TG6) rather than the solitary condition (TS2, TS4, and TS6) (Figure 3c and Table S10), indicating that more energy might be needed at this condition. Notably, gene sets of the cytosolic ribosome, spliceosome, translation, ribosome, and rRNA metabolic process were specifically highly expressed at TG6 (Figure 3d; Table S10); this may suggest that high activity of transcription and translation occurred when older larvae encountered their same instar conspecifics. 

Moreover, the synthesis-related genes of biogenic amines associated with aggressive behavior, including serotonin (5-HT) and dopamine-signaling pathways, were examined. Tryptophan 5-monooxygenase (*TPH*) and tyrosine 3-monooxygenase (*TH*) is the rate-limiting enzyme in 5-HT and dopamine synthesis, respectively. The result showed that *TPH* was not changed and maintained a low level when there was aggressiveness toward conspecifics, whether younger larvae or older larvae (Table S6). On the other hand, two *THs* were identified in the *T. xiaojinensis* genome, one of which was significantly downregulated in TGS2 (Table S7).

All results showed that, compared to the solitary samples (TS2, TS4, and TS6), the conspecific samples (TG2, TG4, and TG6) trended toward higher expression at the respiratory chain-related processes, while cuticle-related processes and metabolism may have interfered, whether younger or older larvae.

#### 3.2.3. Molecular Responses of *T. xiaojinensis* Larvae When Caged with *H. armigera* of Similar Size

**Figure 4:**
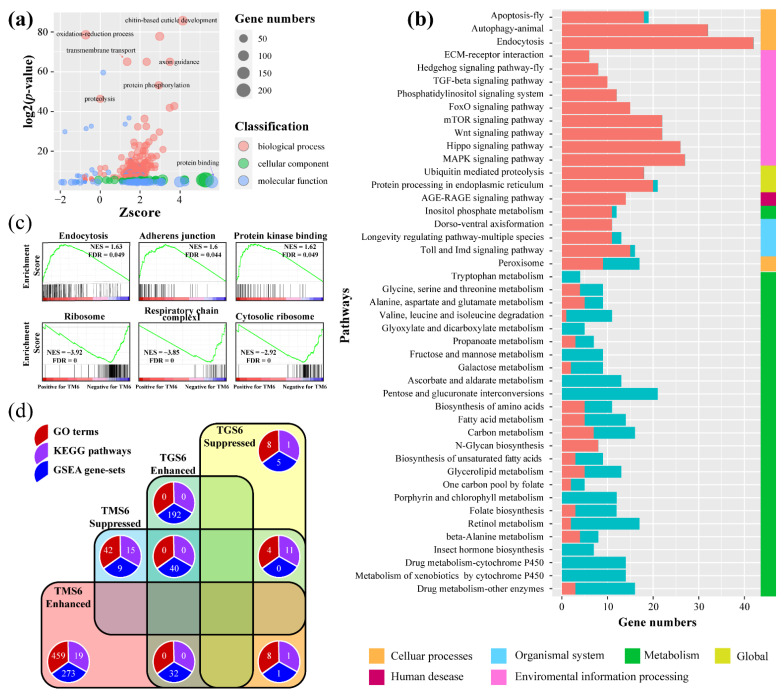
Pathway analysis for TMS6 group. (**a**) GO enrichment analysis for the TMS6 group. The X-axis represents the z-score of the GO terms; the calculation method is presented in the Materials and Methods section. (**b**) KEGG pathway enrichment analysis for TMS6 group. The bar in salmon and turquoise represent the number of upregulated and downregulated genes assigned to the corresponding pathway, respectively. (**c**) GSEA analyses of gene sets for TMS6. NES, normalized enrichment score. FDR, false discovery rate. The top portion of the plot shows the running ES for the gene set as the analysis moves down the ranked list. The bottom portion of the plot shows where the members of the gene set appear in the ranked list of genes. Positive (red) and negative (blue) NES indicate higher and lower expression in TMS samples, respectively. (**d**) Venn diagram illustrating the specific or shared processes (GO terms, KEGG pathways, and GSEA gene sets) of enhanced or suppressed in TGS6 and TMS6. Enhance, GO terms/KEGG pathways enriched by upregulated genes, or GSEA gene sets enriched by genes relatively highly expressed at TM6 or TG6; suppressed, GO terms/KEGG pathways enriched by downregulated genes, or GSEA gene set enriched by genes relatively highly expressed at TS6.

Much more DEGs were identified in L6 *T. xiaojinensis* larva when it showed aggressive behavior toward L5 *H. armigera* larva (which is different from those caged with conspecifics). A total of 512 GO terms were assigned in the TMS6 group (Table S8); 467 GO terms were enriched by upregulated genes, mainly including neuron development processes, such as synapse (GO:0045202), neuromuscular junction (GO:0031594), neuron remodeling (GO:0016322), and neurotransmitter secretion (GO:0007269); cuticle development, such as chitin-base cuticle development (GO:0040003), and structural constituent of chitin-based larval cuticle (GO:0008010) (Figure 4a). A total of 46 GO terms were enriched by downregulated genes, including the oxidation-reduction process (GO:0055114), a metabolic process, such as the fatty acid biosynthetic process (GO:0006633), and a carbohydrate metabolic process (GO:0005975) (Table S8). KEGG analysis was also performed for the TMS6 group; 20 pathways were enriched by upregulated genes (Table S9). Three cellular processes involved in cell fate, transport, and catabolism were significantly enriched, including apoptosis, endocytosis, and autophagy, and cellular process-regulated signaling pathways were enriched, such as mTOR, MAPK, FOXO, and TGF-beta signaling pathways (Figure 4b). Other enriched environmental information processes, such as the Hippo and Hedgehog signaling pathways, were involved in the regulation of organ size, metabolism, and tissue homeostasis. On the other hand, pathways enriched by downregulated genes were mainly from the metabolic processes (97.1%) of carbohydrates, amino acids, cofactors, and vitamins (Figure 4b). Further GSEA results showed that the gene sets, such as positive regulation of lipid metabolic processes, endocytosis, protein kinase binding, establishment of tissue polarity, and adherens junction were highly expressed at TM6. Meanwhile, gene sets related to the ribosome, respiratory chain complex I, cytosolic ribosome, and rRNA binding were lowly expressed (Figure 4c and Table S10). Moreover, *TPH* and *TH* in the synthesis pathways of 5-TH and dopamine were not differentially expressed when *T. xiaojinensis* was aggressive toward *H. armigera*, with low expression (Table S6). 

The comparison of functional enrichment analysis of TMS6 and TGS6 was performed (Figure 4d). GO enrichment analysis showed that the oxidation-reduction process and oxidoreductase activity were downregulated for both comparing groups, while cuticle development-related processes, such as chitin-based extracellular matrix and cuticle development, were upregulated in the TMS6 group but downregulated in the TGS6 group. Pathway analysis showed that metabolisms of carbohydrates, lipids, cofactors, and vitamins were downregulated in both the TMS6 and TGS6 groups. GSEA results exhibited 32 gene sets and were highly expressed in both TM6 and TG6 samples, compared with TS6 samples, such as branching morphogenesis of an epithelial tube, morphogenesis of a branching structure, and morphogenesis of a branching epithelium. Forty other gene sets showed opposite trends in the two compared groups, significantly highly expressed in TG6 but lowly expressed in TM6. Thirty-six out of forty gene sets were associated with the respiratory chain, RNA processes, and ribosome (Table S11).

### 3.3. WGCNA Analysis for T. xiaojinensis

#### 3.3.1. Construction of Gene Co-Expression Network for *T. xiaojinensis*

**Figure 5:**
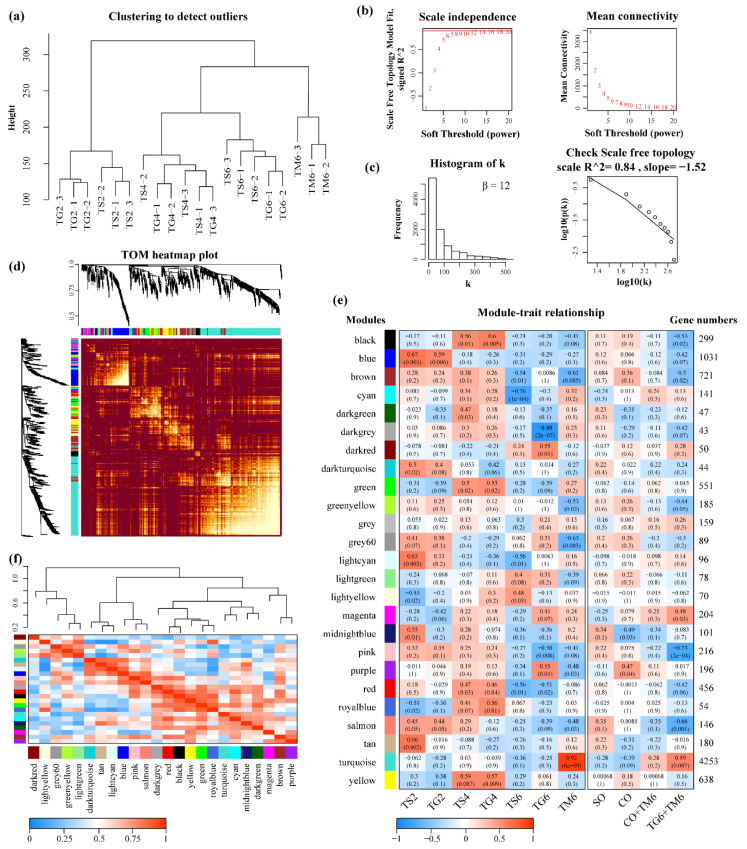
Co-expression network analysis across different compared groups. (**a**) Clustering dendrogram of 20 samples for detecting outliers. (**b**) Analyses of network topology for various soft-thresholding powers (β); the scale-free topology was set as 0.85, which is marked with the red line in the scale independence plot. (**c**) Histogram of connectivity distribution and checking the scale-free topology (R^2^ = 0.84) when β = 12. (**d**) Clustering dendrogram of all expressed genes and visualizing the gene network using a heatmap plot. The longitudinal distance represents the distance between the two genes, and the lateral distance is arbitrary. Different colors represent different modules. The heatmap represents the topological overlap matrix (TOM) among all genes. (**e**) Module–trait relationship. Each row represents a module eigengene, and each column represents a trait (comparing group). The correlation and *p*-value are shown in corresponding cells. The table is colored based on correlation: low to high represents blue through white to red. SO represents the trait for three solitary groups (TS2, TS4, and TS6), while CO represents the trait for three conspecific groups (TG2, TG4, and TG6). (**f**) Cluster analysis of eigengenes and heatmap of connectivity of eigengenes.

We used the WGCNA package tool to construct co-expression networks from 9928 expressed genes (Table S6) in 20 *T. xiaojinensis* samples (Figure 5a) (TG6-3 was identified as an outlier by preliminary construction of the co-expression networks). The soft-thresholding power (β) controlled the independence and average connectivity of the co-expression modules. The topology was roughly more than 0.85 and assigned higher average connectivity when the β value was 12 (Figure 5b). The scale-free topology (R^2^) was 0.84 (β = 12) (Figure 5c). A total of 24 co-expression modules (except for the gray module) were constructed, and the clustering dendrogram of genes is shown in Figure 5d. The module names and the number of genes in corresponding modules are shown in Figure 5e.

#### 3.3.2. Interaction Analysis of Co-Expression Module

The interactions among 24 co-expression modules were analyzed. The TOM heatmap of all genes in the analysis is shown in Figure 5d. The color, ranging from yellow to dark red, indicates low overlap to high overlap. The global difference between the modules is non-significant. This may suggest that the gene expression in different modules is high-scale independent. The correlation coefficiency between module eigengenes and traits was analyzed. The comparing groups were used as traits in this study, including solitary, conspecific, and heterospecific groups. The Turquoise module was significantly positively associated with TM6 (4253 genes, cor = 0.92, *p*-value = 8 × 10^−9^), while the module that was highly negatively correlated to TG6 was the DarkGrey module (43 genes, cor = −0.88, *p*-value = 2 × 10^−7^) (Figure 5e). No specific module was significantly associated with other groups, but several modules were associated with both solitary and conspecific groups, such as the Blue and DarkTurquoise modules for TG2 and TS2, Black, Green, and Yellow modules for TG4 and TS4, implying that limited specific changes were found between solitary and conspecific groups at L2 or L4 *T. xiaojinensis* larvae. To find the interaction between these constructed co-expression modules, cluster analysis was performed on these eigengenes to examine the connectivity among modules (Figure 5f), which could be divided into two clusters, including five modules (DarkRed, LightYellow, Grey60, GreenYellow, and DarkTurquoise) and the remaining 19 modules, indicating that the connectivity effect between modules is significantly different.

#### 3.3.3. Functional Enrichment Analysis and Hub Genes Identification in Turquoise Module

**Figure 6:**
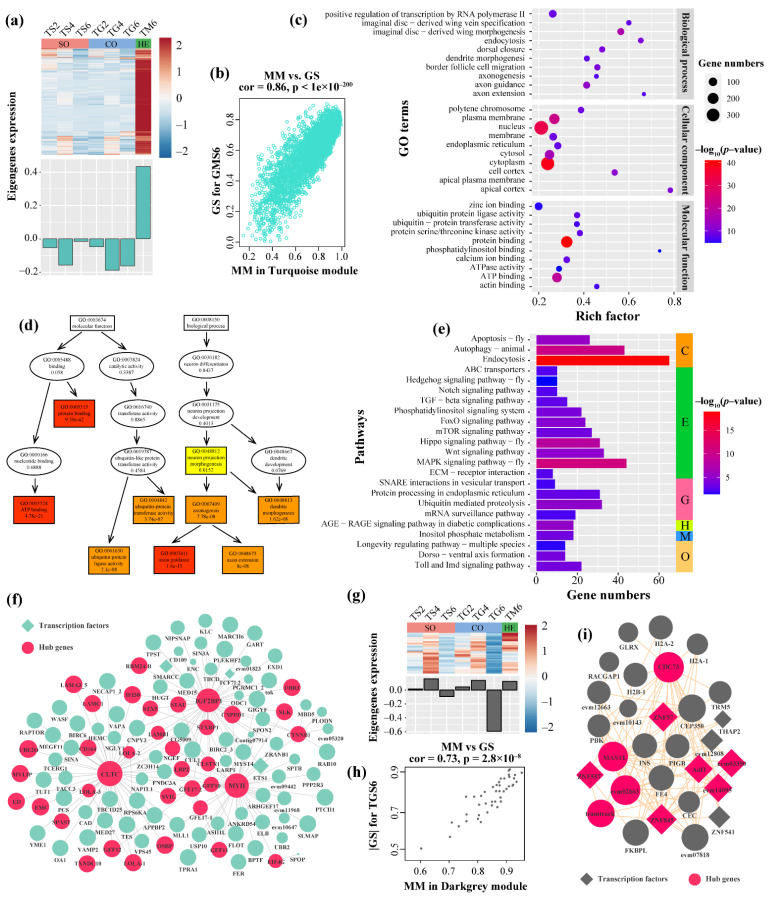
Turquoise and DarkGrey modules analyses. Gene expression pattern of Turquoise (**a**) and DarkGrey (**g**) modules. The heat map shows the expression patterns of each gene contained in the corresponding module; upregulated and downregulated genes are represented by red and blue, respectively. The histogram shows the variation in module eigengenes expressed in different samples. SO, solitary groups; CO, conspecific groups; HE, heterospecific groups. Scatter plots for correlations between module membership (MM) and gene significance (GS) in the Turquoise (**b**) and DarkGrey (**h**) modules. (**c**) GO and (**e**) KEGG enrichment for central genes (MM > 0.8 and GS > 0.6) from Turquoise module. (**d**) The thumbnail view of the directed acyclic graph (DAG) on biological processes and molecular function. The network for top 150 connectivity genes in the Turquoise (**f**) and DarkGrey (**i**) module. A node with a larger size indicates higher intramodular connectivity. The full list of the gene names of the nodes is shown in Table S12.

The Turquoise module is a trait module for the response of *T. xiaojinensis* caged with *H. armigera*, which contained 4253 genes (Table S12). We performed gene trend expression analysis of the Turquoise module, which was relatively highly expressed only in the TM6 group (Figure 6a). The correlation between module membership (MM) and gene significance (GS) of the Turquoise module is depicted as a scatter plot in Figure 6b. To better annotate the module function, we first selected 1922 genes whose MM > 0.8 and GS > 0.6 as central genes (Table S13). GO enrichment analysis for central genes showed enrichment for several GO terms that were functionally associated with neuron development, such as the BP of axon guidance, axon extension, and dendrite morphogenesis, and related to the MF of the ubiquitin protein transferase activity, and protein and ATP binding activity (Figure 6c,d and Table S14). Moreover, KEGG pathway analysis showed that the Turquoise module was significantly enriched in 23 pathways, including cellular processes such as apoptosis, endocytosis, and autophagy, and environmental information processes, such as MAPK, Hedgehog, mTOR, Hippo, and FOXO signaling pathways (Figure 6e and Table S14). 

**Figure 7:**
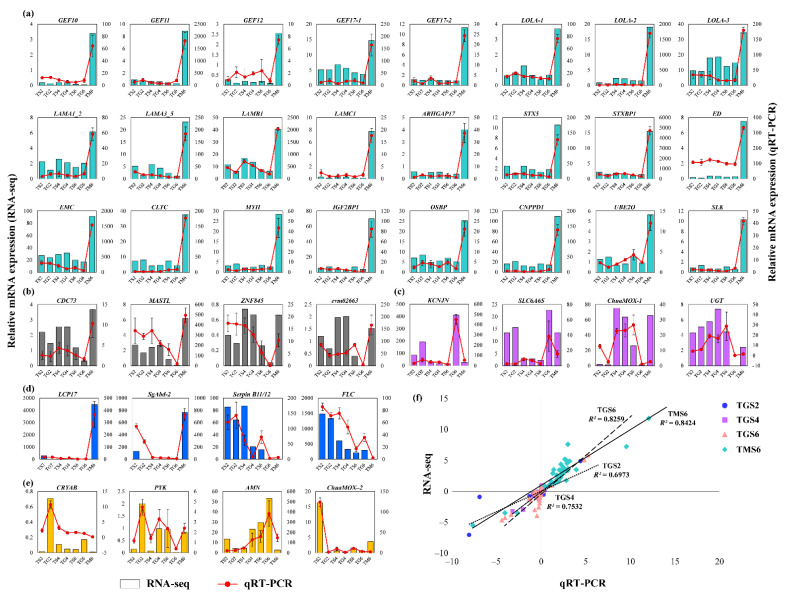
Validation of the selected genes in *T. xiaojinensis* from RNA-seq by qRT-PCR. RNA-seq and qRT-PCR results of hub genes from (**a**) Turquoise and (**b**) DarkGrey modules, DEGs from (**c**) TGS6 and (**d**) TMS6, as well as (**e**) four randomly selected genes. (**f**) Comparison of the log_2_ of gene expression ratios between RNA-seq data and qRT-PCR results. Cycle, square, triangle, and rhombus represent the results for TGS2, TGS4, TGS6, and TMS6, respectively.

Through further analysis of this module, several hub genes were defined (Table S13), such as clathrin heavy chain (*CLTC*), myosin heavy chain (*MYH*), and insulin-like growth factor 2 mRNA-binding protein 1 (*IGF2BP1*) (Figure 6f and Table S13). Among these hub genes, 17 (34%) were reported in *D. melanogaster*, such as syntaxin 5 (*STX5*), laminin A (*LAMA*), and echinoid (*ED*) (Table S13). The mRNA expression of part of these hub genes was confirmed by qRT-PCR (Figure 7a). Furthermore, several transcription factors had high connectivity in the Turquoise module, including the zinc finger MIZ domain-containing protein (*ZMIZ*), zinc finger protein 676 (*ZNF676*), zinc finger protein 418 (*ZNF418*), and zinc finger protein 853 (*ZNF853*); these TFs may play regulated roles when *T. xiaojinensis* larvae are aggressive toward *H. armigera*.

#### 3.3.4. Functional Enrichment Analysis and Hub Genes Identification in the DarkGrey Module

The DarkGrey module only contained 43 genes, which was significantly negatively associated with TGS6, showing that these genes specifically had low expression in L6 *T. xiaojinensis* larvae when aggressive toward L6 conspecifics. A total of 29 genes were identified as central genes (MM > 0.8, |GS|> 0.6), which were further used to perform GO and KEGG enrichment, BP of positive regulation of mitotic cell cycle, CC of nuclear nucleosome, and MF of protein heterodimerization activity; DNA binding was significantly enriched in this module (Table S15). 

The eigengenes expression analysis of the DarkGrey module was consistent with the heatmap result (Figure 6g,h). A total of nine (31%) genes from the central genes in this module were transcription factors (TFs), including transcription factor Adf-1 (*ADF-1*), which is involved in regulating neural activity. The expressions of another three unknown MADF-domain-containing TFs (evm03390, evm14095, and evm12808) was highly correlated with *ADF-1* (Figure 6i), implying that these MADF-domain-containing TFs may have similar functions associated with *ADF-1*. The remaining were zf-C2H2 and THAP domain-containing TFs, such as zinc finger protein 557 (*ZNF557*), zinc finger protein 57 (*ZNF57*), and zinc finger protein 845 (*ZNF845*). These TFs had low expression at TG6. In addition to the above TFs, several hub genes were identified from this module; parafibromin (*CDC73*) participated in histones, ubiquitination, and several histone H2A-1, H2A-2, and H2B, had co-expression with *CDC73* (Figure 6i), which had low expression at TG6. Other hub genes in the DarkGrey module are shown in Table S13.

### 3.4. Experimental Validation

To validate the veracity and reliability of the DEGs identified by RNA-seq, 39 genes were selected for qRT-PCR validation from the Turquoise module, DarkGrey module, DEGs from TGS6, and TMS6, as well as randomly selected genes (Figure 7a–e). qRT-PCR data significantly correlated with the RNA-seq result, with a correlation coefficient of 0.7 (for TGS2), 0.75 (for TGS4), 0.83 (for TGS6), and 0.84 (for TMS6) (Figure 7f), demonstrating the credibility of the transcriptome results.

## 4. Discussion

*T. xiaojinensis* is distributed naturally in the mountainous regions (altitudes: 3600–4800 m) of the Tibetan Plateau, the larvae of which are the host of the entomopathogenic fungus *Ophiocordyceps sinensis* [58]. This caterpillar fungus complex, named Chinese cordyceps, is regarded as a traditional Chinese medicine and is well-known for its immunomodulatory activities [59]. Artificial cultivation of the caterpillar fungus complex is urgently needed to ensure its protection as a bio-resource and for commercial supply [34]. One of the crucial steps in the artificial cultivation of this complex is the artificial rearing of the insect hosts [34]. The larvae of *T. xiaojinensis* reared in the laboratory were highly aggressive toward conspecifics. Aggressive behaviors are pervasive throughout the animal kingdom. Aggression is beneficial in helping animals to compete for limited resources, such as food and mates. However, aggressive encounters can also lead to physical damage, and in some instances, even death, causing large-scale rearing difficulties for aggressive insects [60,61]. 

### 4.1. Influences of Food on Aggressive Behavior

Food intake is the most important behavior for the larval stage. Despite the density-dependent factor, the propensity for aggressiveness may also be influenced by many density-independent factors, one of which is limited in the amount or quality of food [62]. For example, aggressive behavior and cannibalism of *H. armigera* [63] and *Henosepilachna pustulosa* [64] may arise when food is in short supply or of low quality, due to asynchrony with host plants, or when encouraged by abiotic environmental factors. The aggressive behavior of *T. xiaojinensis* larvae—different from the general rule mention above—does not seem sensitive as to whether food is provided, showing a similar ABS score and survival rate in the state of the feeding or starved condition. The differences could be due, at least in part, to *T. xiaojinensis* larvae having a very strong hunger tolerance, i.e., they could survive for at least seven days without food (unpublished data), and in part due to different predation behavior. Furthermore, the *T. xiaojinensis* attacker will rarely chase the victim after hitting (or biting) it; it seems as if they fight for space rather than food resources.

### 4.2. Transcriptional Response When T. xiaojinensis Showed Aggressiveness toward Conspecifics

In the present study, although a higher survival rate occurred in younger larvae (L2–L2), a similar ABS score was observed among the same instar groups (L2–L2, L4–L4, and L6–L6), exhibiting a highly aggressive trend toward the same instar stage larvae of conspecific. 

Oxidative phosphorylation (OXPHOS) is the metabolic pathway in which cells use enzymes to oxidize nutrients and produce adenosine triphosphate (ATP). Reducing the activity of OXPHOS was positively correlated with aggressive behavior in honey bees [65,66]. In mice, aggressive behavior resulting from social stress would selectively reduce COX expression and activity, compromising energy demand, with consequence for cognition and behavior [67]. According to the GSEA result, OXPHOS has a high NES value in the TGS2, TGS4, and TGS6 groups (different from honey bees and mice). When *T. xiaojinensis* fought, *COX4*, *COX5A*, *COX6C*, and *COX7C*, were slightly upregulated in *T. xiaojinensis*. Similar results were found in the subunit of complex I in OXPHOS, such as NADH dehydrogenase 1 alpha (*NDUFA2*, *NDUFA5*, *NDUFA6*, and *NDUFA8*) and NADH dehydrogenase 1 beta (*NDUFB5*, *NDUFB7*, and *NDUFB9*), indicating that more energy was needed while aggressive behavior occurred.

Biogenic amines play important roles in modulating aggressive behavior; a low level of serotonin (5-HT) could increase the level of aggression and impulsivity in vertebrates [68]; for example, increasing levels of 5-HT using 5-HT precursors, 5-HT reuptake inhibitors, and 5-HT_1A_, and 5-HT_1B_ receptor agonists reduces aggressive behavior in rodents [69,70,71,72]. On the other hand, 5-HT also mediates aggressive behavior in lobsters and crayfish, but the effects of the serotonergic system in invertebrates are opposite to those in vertebrates [19,73,74]. *TPH* is the rate-limiting enzyme in 5-HT synthesis, catalyzing the tryptophan to 5-hydroxy-L-tryptophan (5HTP), which plays a key role in aggressive disposition [75,76]. In *T. xiaojinensis*, the mRNA expression of *TPH* was not changed and maintained a low level when caged with conspecifics, whether younger larvae or older larvae (Table S6). This implies that the *TPH* gene may play a limited role in aggressive behavior in *T. xiaojinensis* larvae toward conspecifics. Dopamine is another biogenic amine whose aberrant signaling plays a pivotal role in the ontogeny of aggressive behavior [77]. In vertebrates, dopamine levels were changed in the opposite direction to serotonin in the process of aggressive behavior [78]. *TH* is a rate-limiting enzyme in dopamine synthesis, catalyzing the L-tyrosine to L-dopa. Two *THs* were identified in *T. xiaojinensis*, one of which was significantly downregulated in TGS2 (Table S7), suggesting that decreasing levels of dopamine may be associated with aggressive behavior in younger *T. xiaojinensis* when confronting conspecifics, which is different from the rat and human [18,78]. It seems that biogenic amines play different roles in regulating aggressive behavior in different species.

Other shared suppressed processes in *T. xiaojinensis* when caged with conspecifics were associated with cuticle development; the genes, such as cuticle protein (*CP3*, *CP7*, *CP8*, and *CP19*), endocuticle structural glycoprotein (*ABD-5*, *SgAbd-1*, and *SgAbd-2*), flexible cuticle protein 12 (*CP12*), larval cuticle protein (*A1A*, *A2B*, *A3A*, and *LCP17*), and pupal cuticle protein (*PCP36a*) were significantly downregulated at TGS2, and slightly downregulated at TGS4 and TGS6 (Table S7). It seems that aggressive behaviors toward conspecifics may interfere with the structural integrity.

The results show that, when *T. xiaojinensis* showed aggressive behavior toward the same instar conspecific, limited genes were significantly differentially expressed, especially the older larvae. Higher activity of OXPHOS, but lower activity of biogenic amines, may have been needed when there was aggressive behavior of *T. xiaojinensis* toward conspecifics; meanwhile, cuticle development and metabolism may have interfered.

### 4.3. Transcriptional Response When T. xiaojinensis Showed Aggressiveness toward Heterospecific

We also compared the aggressive behavior of L6 *T. xiaojinensis* larvae when caged with conspecifics and heterospecifics (*H. armigera*). The ABS scores of both insect larvae, when caged with heterospecifics, were lower than those when caged with the same instar conspecifics, while the survival rate was higher when caged with heterospecifics. This would suggest that both insects have higher aggressiveness toward same instar conspecifics. On the transcriptional level, when *T. xiaojinensis* showed aggressive behavior toward *H. armigera* (TMS6), different from the limited DEGs from the aggressiveness toward their conspecific (TGS6), much more DEGs were identified. In particular, the upregulated genes in neurons, apoptosis, endocytosis, autophagy, and some other pathways regulated the body size and tissue homeostasis. These results taht suggest there were great differences in the transcription level of *T. xiaojinensis* when aggressive toward conspecifics and heterospecifics.

Much of the work on the neurobiology of aggressive behavior establishes the role of neurotransmitters in mediating and modulating levels of aggression [9]. In this study, neuron-related processes were significantly enhanced when subjects were aggressive toward *H. armigera*, such as neurogenesis, neurotransmitter secretion, synapse, synaptic vesicle, and neuronal cell body. The genes shared in these processes, including the Ras-related protein (*Rab-3C*, *Rab-6A*, *Rab-7A*, *Rab-10*, *Rab-14*, *Rab-35*, and *Rab-39B*), exocyst complex component (*exoc4* and *exoc8*), myosin (*MYO*), and syntaxin 1A (*STX1A*), were significantly upregulated in the TMS6 group, but did not respond in the TGS6 group (Table S7). This result suggests that neuron-related processes play different roles when *T. xiaojinensis* is aggressive toward different targets.

In particular, for both of the dopamine synthesis genes, *THs* were not differentially expressed when *T. xiaojinensis* was aggressive toward *H. armigera*, with low expression. It seems that the dopamine synthesis gene *TH* plays different roles in modulating the aggressive behavior of *T. xiaojinensis* when caged with conspecifics and heterospecifics. Similarly, for the rate-limiting enzyme in 5-HT synthesis, *TPH* is not differentially expressed, and maintains a low expression level when caged with *H. armigera*. This suggests that the *TPH* gene may play a limited role in aggressive behavior in *T. xiaojinensis* larvae, whether toward conspecifics or heterospecifics. Additionally, cuticle development processes were significantly enhanced when *T. xiaojinensis* was aggressive toward *H. armigera*. The upregulated cuticle-related gene included part of the gene mentioned above, such as *CP3*, *CP7*, *CP8*, *CP19*, *SgAbd-1*, *SgAbd-2*, *CP12*, *A1A*, *A3A*, and *LCP17*. Applying a large amount of cuticle protein synthesis may thicken the cuticle surface to increase defense against *H. armigera*.

*T. xiaojinensis* never meet *H. armigera* in the wild; this is the first time that they met in the laboratory. *T. xiaojinensis* larvae may feel threatened by the unknown aggressive insect; therefore, a large number of genes were differentially expressed in response to the stress. In general, when *T. xiaojinensis* was aggressive toward *H. armigera*, many basal processes related to cell fate, neuron development, body size, tissue homeostasis, and cuticle development were enhanced, but plenty of metabolisms were significantly suppressed.

### 4.4. Hub Genes Modulating Aggressive Behavior

WGCNA was widely used in insects in recent years. In *Nilaparvata lugens*, WGCNA was used to analyze the embryogenesis; hub genes at each embryonic stage with possible crucial roles were identified. In suppression of the mRNA expression of hub gene *MSTRG.3372*, the embryo was indeed observed as abnormal [79]. To identify key genes of the insecticide resistance of *Spodoptera litura*, WGCNA was performed. Eight genes coding UDP-glucuronosyltransferases were determined to be the hub genes in insecticide resistance [80], which proved essential for the elimination and detoxification of drugs, xenobiotics, and chemical compounds [81,82]. In the study of *Ericerus pela*, the hub genes associated with cold resistance were identified by using WGCNA; some well-studied cold resistance-related genes, such as heat shock protein, and antifreeze protein, could be rediscovered by this method [83]. Through these studies, it was confirmed that many of the hub genes obtained by WGCNA analysis were indeed very important genes; the WGCNA method is reliable. We also performed WGCNA to obtain the hub genes related to aggressive behavior in *T. xiaojinensis*.

Correlation analysis between the co-expression module and traits was carried out, and two highly significant trait-related modules (|cor| > 0.75, *p*-value < 1 × 10^−5^) were identified. The Turquoise module was significantly positively related to TM6. Functional enrichment showed that the central genes of the Turquoise module were associated with cellular processes and their regulating pathways, and particularly, neuron development. Several hub genes were associated with neuron development, such as *STX5*, and its binding protein syntaxin-binding protein (*STXBP*). *STX5* was potentially involved in the docking of synaptic vesicles and neurotransmitter secretion [84], also considered to be a candidate gene affecting aggressive behavior in *D. melanogaster* [29]. Previous studies reported that Rho guanine nucleotide exchange factor 64C (*GEF64C*) played a role in axon guidance [85], and P-element insertions in *GEF64C* were associated with decreased levels of aggression in *D. melanogaster* [9]. In our study, six *GEF* were identified as hub genes, such as neuronal guanine nucleotide exchange factor (*NGEF*), e.g., GEF64C, which play a role in axon guidance, regulating ephrin-induced growth cone collapse and dendritic spine morphogenesis [86]. The other five hub genes *GEF10*, *GEF11*, *GEF12,* and two *GEF17s* participated in myelination in the peripheral nervous system and neurotrophin-induced neurite outgrowth [87,88]. Three longitudinals lacking (*LOLA*) proteins are hub genes in the Turquoise module as well. *LOLA* is a TF required for axon growth and guidance in the nervous system [89], its function in modulating aggressive behavior was also confirmed by a previous study in *D. melanogaster* [9]. Similarly, nervous system development-related hub genes *ED*, *LAMA,* and extramacrochaetae (*EMC*); were reported to be associated with aggressive behavior in *D. melanogaster* [9], but the regulating effect of *ED* and *LAM* seems to be the opposite of *T. xiaojinensis*. The expression of all hub genes mentioned above was confirmed by qRT-PCR (Figure 7a). In general, at least 34% of the hub genes identified in the Turquoise module were proven to play a role in modulating aggressive behavior in *D. melanogaster*, suggesting the reliability of our results. The hub genes or even the central genes filtered from the Turquoise module were important to the aggressiveness in *T. xiaojinensis*, and they are worthy of further studies. 

## 5. Conclusions

*T. xiaojinensis* is a common species found in the mountainous regions of the Tibetan Plateau, and it is highly aggressive toward conspecifics. High-throughput RNA-seq with a reference genome provides opportunities for in-depth analysis when *T. xiaojinensis* are aggressive toward conspecifics and heterospecifics. This study provided a set of important pathways and DEGs associated with aggressive behavior, and the weighted gene co-expression network for traits was constructed; the central and hub genes involved in aggressive behavior were also obtained. The results revealed molecular responses when *T. xiaojinensis* showed aggressiveness toward conspecifics and heterospecifics. These data are important for better understanding the aggressive behavior of Lepidopteran larvae at the transcriptional level and provide a theoretical basis for further analysis of the genetic mechanism of the insect’s aggression.

## Data Availability

All of the raw sequence data were deposited in the NCBI Sequence Read Archive (SRA) under BioProject accession number PRJNA657440.

## Supplementary Materials

The following are available online at https://www.mdpi.com/article/10.3390/insects12070577/s1, Figure S1: Insect combinations of the assays, Figure S2: Food consumption rate of *T. xiaojinensis*, Figure S3: ABS for three different aggressive behaviors, Table S1: Primers used for qRT-PCR, Table S2: Statistical analysis of aggressive behavior in *T. xiaojinensis*, Table S3: Scores for three aggressive behaviors and statistical analysis, Table S4: Survival analysis of *T. xiaojinensis* caged with conspecifics and heterospecific, Table S5: Summary of RNA-seq results, Table S6: FPKM value of expressed genes in *T. xiaojinensis*, Table S7: DEGs in *T. xiaojinensis* under different comparing groups, Table S8: GO enrichment analysis for *T. xiaojinensis*, Table S9: KEGG enrichment analysis for *T. xiaojinensis*, Table S10: GSEA for *T. xiaojinensis*, Table S11: Enrichment comparison for TGS6 and TMS6, Table S12: Genes list for Turquoise and DarkGrey module, Table S13: Central and hub genes in Turquoise and DarkGrey module, Table S14: GO and KEGG enrichment analysis for Turquoise module, Table S15: GO and KEGG enrichment analysis for DarkGrey module.
