# Supplementary material for: Transcriptome Analyses Provide Insights into the Aggressive Behavior toward Conspecific and Heterospecific in Thitarodes xiaojinensis (Lepidoptera: Hepialidae)
Source: Insects. 2021 Jun 25;12(7):577. doi: 10.3390/insects12070577 (PMC8306418; doi:10.3390/insects12070577)
Supplement: Supplementary file 1 [file insects-12-00577-s001.zip › Supplementary Figure Legend.pdf]

Figure S1: Insect combinations of the assays. Including L2vsL2 (L2-L2 comparing group), L2vsL4 (L2-L4 and L4-L2), L2vsL6 (L2-L6 and L6-L2), L4vsL4 (L4-L4), L4vsL6 (L4-L6 and L6-L4), L6vsL6 (L6-L6, shared in assay 1 and assay 2), L6vsL5 (L6-L5 and L5-L6), and L5vsL5(L5-L5). The scored larva in L2vsL2, L4vsL4 and L6vsL6 was marked with dark point (indicated with red circle in the picture).

Figure S2: Food consumption rate of *T. xiaojinensis*. Food consumption rate of **(a)** L2, **(b)** L4 and **(c)** L6 *T. xiaojinensis* after 1 day, 3 days and 7 days starvation.

Figure S3: ABS for three different aggressive behaviors. Mean aggression behavior scores (ABS) (±SE) of different comparing groups with food **(a)** or without food **(b)**. blue, orange, and grey bar represented "hit", "bite" and "chase" behavior, respectively.
