# Supplementary figures and images for: Transcriptome Analyses Provide Insights into the Aggressive Behavior toward Conspecific and Heterospecific in Thitarodes xiaojinensis (Lepidoptera: Hepialidae)
Source: Insects. 2021 Jun 25;12(7):577. doi: 10.3390/insects12070577 (PMC8306418; doi:10.3390/insects12070577)

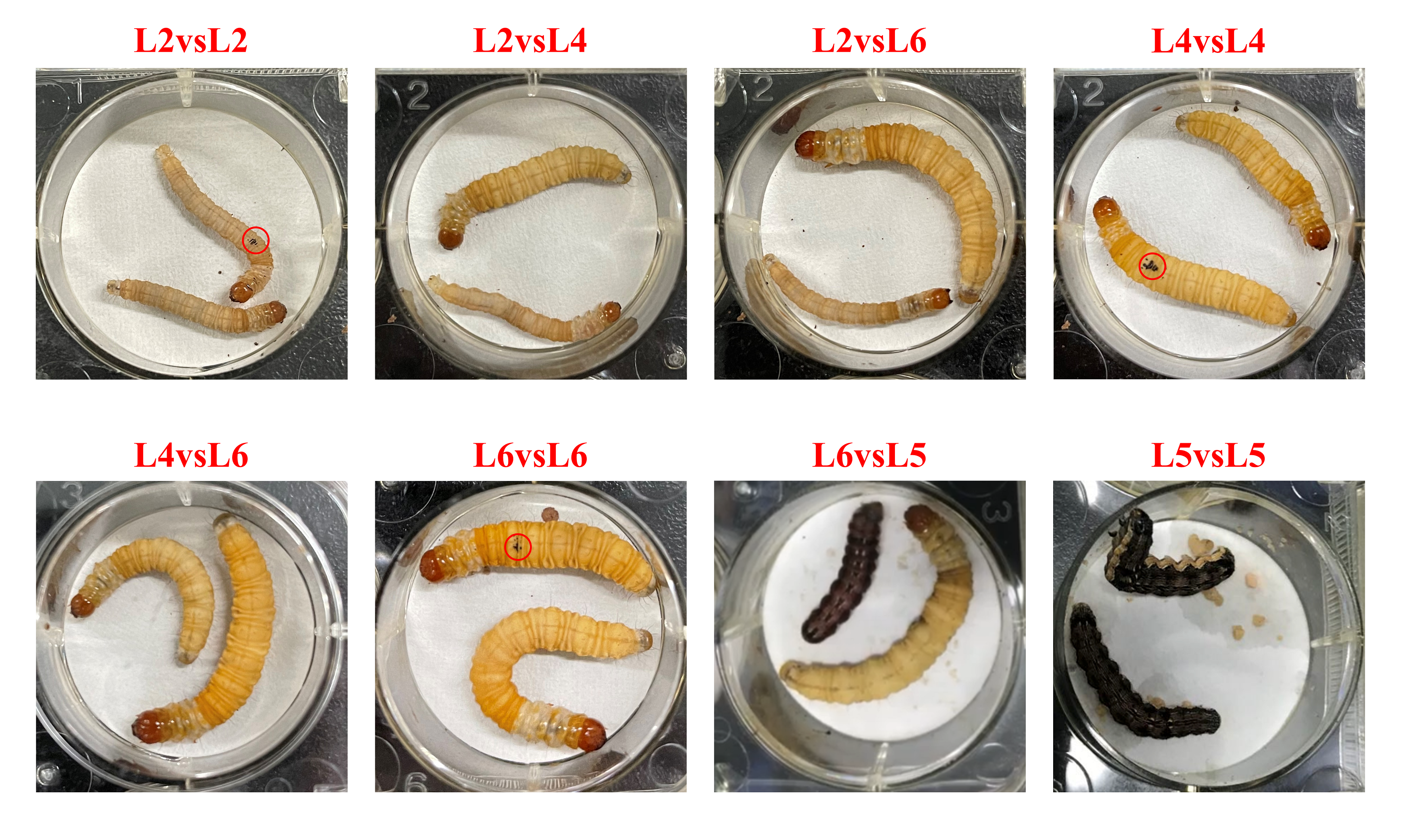

Supplement: Supplementary file 1 [file insects-12-00577-s001.zip › Figure S1.tif]

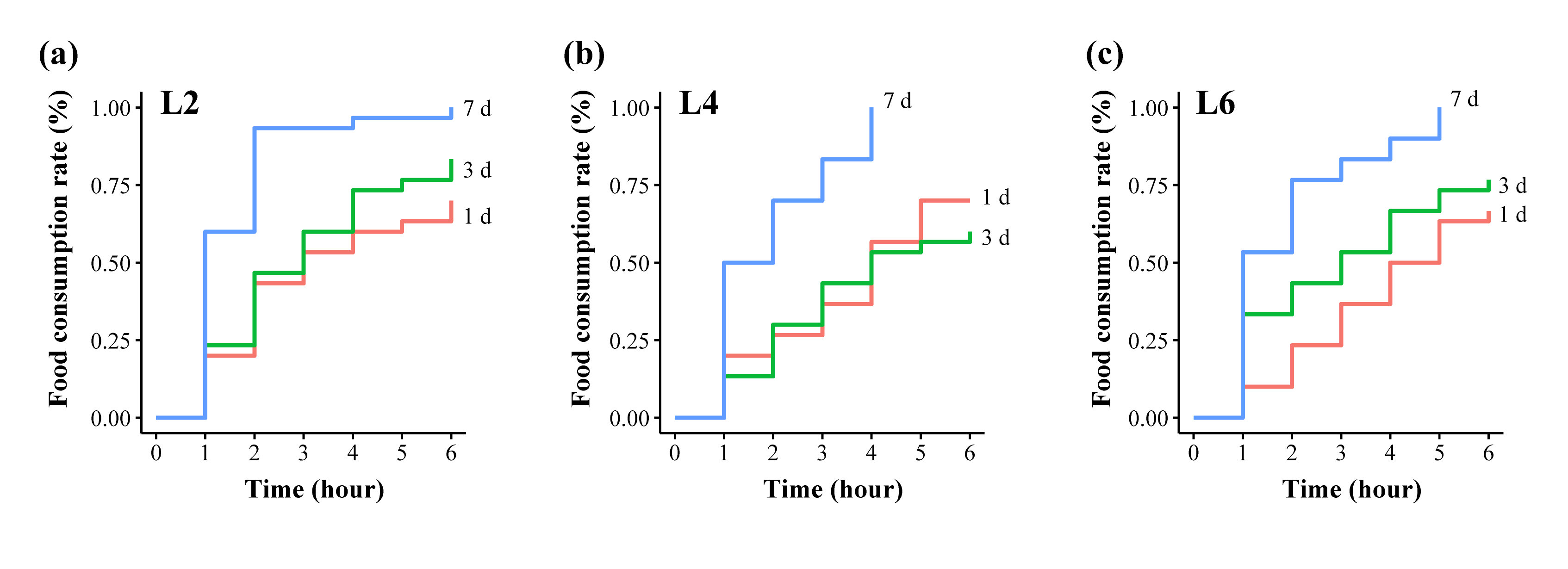

Supplement: Supplementary file 1 [file insects-12-00577-s001.zip › Figure S2.tif]

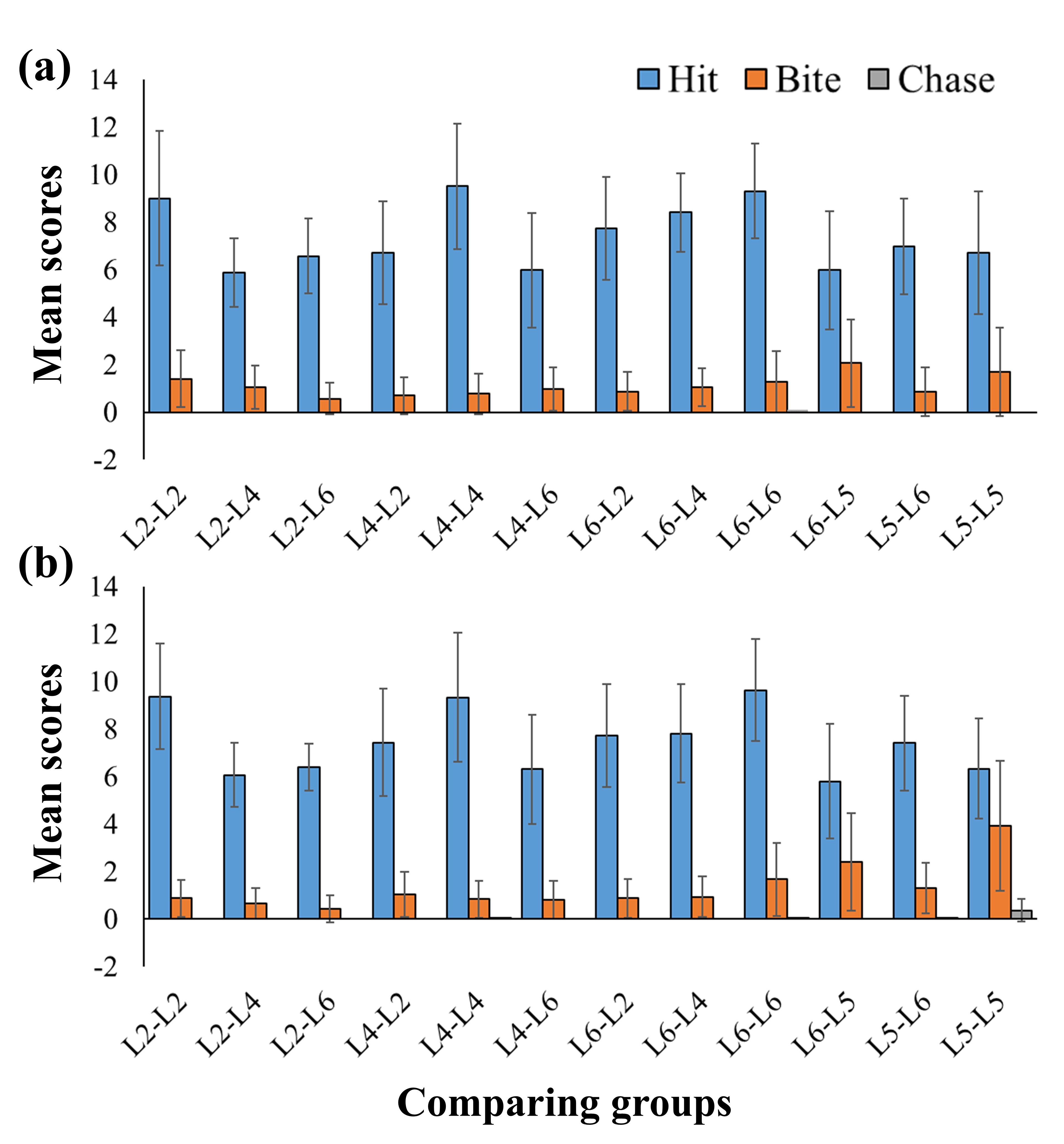

Supplement: Supplementary file 1 [file insects-12-00577-s001.zip › Figure S3.tif]
